# fastGxE: Powering genome-wide detection of genotype-environment interactions in biobank studies

**DOI:** 10.21203/rs.3.rs-5952773/v1

**Published:** 2025-03-20

**Authors:** Chao Ning, Xiang Zhou

**Affiliations:** 1 Department of Biostatistics, University of Michigan, Ann Arbor, MI 48109, USA; 2 Center for Statistical Genetics, University of Michigan, Ann Arbor, MI 48109, USA

## Abstract

Traditional genome-wide association studies (GWAS) have primarily focused on detecting main genotype effects, often overlooking genotype-environment interactions (GxE), which are essential for understanding context-specific genetic effects and refining disease etiology. Here, we present fastGxE, a scalable and effective genome-wide GxE method designed to identify genetic variants that interact with environmental factors to influence traits of interest. fastGxE controls for both polygenic effects and polygenic interaction effects, is robust to the number of environmental factors involved in GxE interactions, and ensures scalability for genome-wide GxE analysis in large biobank studies, achieving speed improvements of 32.98–126.49 times over existing approaches. We illustrate the benefits of fastGxE through extensive simulations and an in-depth analysis of 32 physical traits and 67 blood biomarkers from the UK Biobank. In real data applications, fastGxE identifies nine genomic loci associated with physical traits, including six novel ones, and 26 genomic loci associated with blood biomarkers, 19 of which are novel. The new discoveries highlight the dynamic interplay between genetics and the environment, uncovering potentially clinically significant pathways that could inform personalized interventions and treatment strategies.

## Introduction

Genome-wide association studies (GWAS) have successfully identified many genetic variants associated with diseases and disease-related phenotypes, reshaping our understanding of the genetic architecture underlying complex traits. Despite these successes, most GWAS have focused on detecting variants with genetic main effects on the trait, implicitly assuming a constant effect size across diverse environmental contexts. This traditional approach overlooks an important aspect of genetic influence — namely, that genetic effects can be context-dependent and modulated by environmental factors^1,2^. Indeed, both genetic and environmental factors are major contributors to phenotypic variation, and their interactions play a pivotal role in shaping complex human traits^3,4^. For example, the genetic predisposition to obesity may manifest differently depending on lifestyle factors such as diet and physical activity^5^. As a result, understanding these gene-environment interactions (GxE) is crucial, as they reveal how genetic risk manifests across different environmental exposures, highlight context-specific genetic effects, and ultimately refine our understanding of disease etiology. The recent availability of large-scale biobank data has provided an unprecedented opportunity to systematically characterize GxE at a scale and resolution previously unattainable. Leveraging these datasets, GxE studies have started to dissect the dynamic interplay between genes and the environment^6^, uncover context-specific genetic effects^7^, generate deeper insights into the architecture of complex traits^8^, build more accurate models for genetic risk prediction by incorporating GxE effects^9^, and ultimately pave the way for personalized medicine strategies that integrate both genetic and environmental factors^10,11^.

Despite the promise of GxE studies, conducting effective genome-wide GxE association analysis in biobank scale studies presents several important statistical and computational challenges. Two primary strategies have been developed for detecting GxE associations. The first strategy focuses on single-environment GxE tests, using tools such as the linear regression model framework implemented in PLINK^12^ and the linear mixed model framework implemented in fastGWA-GE^13^. These approaches directly assess interaction between genetic variants and a single environmental factor. However, they often lack sufficient statistical power, even in large-scale biobank data, and have sometimes failed to detect well-established GxE loci, such as the *FTO* locus for body mass index (BMI)^13^. An alternative strategy involves joint GxE testing across multiple environmental factors, as implemented in tools like StructLMM^14^ and GEM^15^. These multi-environment GxE approaches offer much improved statistical power compared to single-environment GxE tests, as genetic variants often interact with multiple environmental factors to influence phenotypes^6,14^. However, these approaches have three key limitations. First, their statistical power can be substantially reduced when only a small subset of environments contributes to GxE interactions. Second, they are computationally intensive, restricted to analyzing candidate genomic regions, and lack the scalability required for genome-wide GxE analysis in biobank-scale datasets. Third, these approaches do not account for polygenic effects or polygenic interaction effects contributed by genome-wide variants. As we will demonstrate here, both types of polygenic effects explain a substantial proportion of phenotypic variance, and ignoring them have consequences similar to neglecting population stratification — a well-known source of confounding that increases the risk of false positive findings in association analyses^16^. Unfortunately, accounting for polygenic effects and polygenic interaction effects presents substantial computational challenges, particularly in the context of multi-environmental GxE approaches.

Here, we present fastGxE, a scalable and effective genome-wide multi-environment GxE method designed to identify genetic variants that interact with one or multiple environmental factors. fastGxE controls for both polygenic effects and polygenic interaction effects, is robust to the number of environmental factors contributing to GxE interactions, and ensures scalability for genome-wide analysis in large biobank studies, achieving speed improvements of 32.98–126.49 times over existing approaches. We also developed mmSuSiE, an extension of SuSiE specifically designed for mixed-model analysis, to identify the environmental factors driving the detected GxE interactions by fastGxE. We illustrate the benefits of fastGxE through extensive simulations and an in-depth analysis of 32 physical traits and 67 blood biomarkers from the UK Biobank. In real data applications, fastGxE identifies nine genomic loci associated with physical traits, including six novel ones, and 26 genomic loci associated with blood biomarkers, 19 of which are novel. Example of interactions include *CHRNA5-A3-B4* interacting with smoking status, time spent watching television and water intake to influence FEV1/FVC ratio (FFR), *FBXO32* interacting with age to influence pulse pressure, and *ARHGEF3* interacting with age, time spent watching television and water intake to be associated with platelet distribution width. These new discoveries highlight the dynamic interplay between genetics and the environment, uncovering potentially clinically significant pathways that could inform personalized interventions and treatment strategies.

## Results

### Runtime comparison

**Figure 1.**
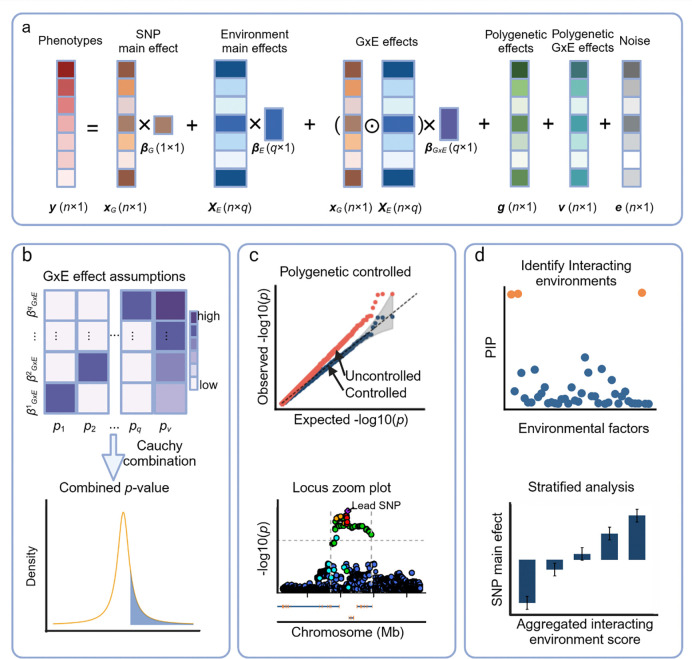
Schematic overview of fastGxE. fastGxE is a scalable and effective method designed for genome-wide GxE association analysis. fastGxE handles multiple environmental factors and examines one SNP at a time, decomposing the phenotype into SNP main effect, environmental main effects, GxE interaction effects, while controlling for both polygenic effects and polygenic interaction effects (a). fastGxE evaluates various GxE effect size configurations and combine the resulting *p*-values into a single *p*-value to test whether the SNP interacts with at least one environmental factor (b). With controlled polygenic and polygenic interaction effects, fastGxE generates calibrated *p*-values for identifying candidate GxE loci (c). Additionally, it utilizes mmSuSiE, an extension of the SuSiE algorithm, to identify the environmental factors driving the detected GxE interactions and employs the stratified Wald test to validate and visualize these interactions (d). Figure is created with BioRender.com. y is an *n*-vector of phenotypic measurements; $x_{G}$ is an n-vector of genotypes for the SNP of focus; $X_{E}$ is an n by q matrix for q measured environmental factors; $\beta_{G}$ is the genetic main effect size; $\beta_{E}$ is an q-vector of environment main effect sizes; $\beta_{GXE}$ is an q-vector of interaction effects between the SNP of focus and q environmental factors; ⊙ denotes the Hadamard product; g is a n-vector of polygenetic effects; v is a n-vector of polygenetic GxE effects; e is a n-vector of residual errors.

**Figure 2.**
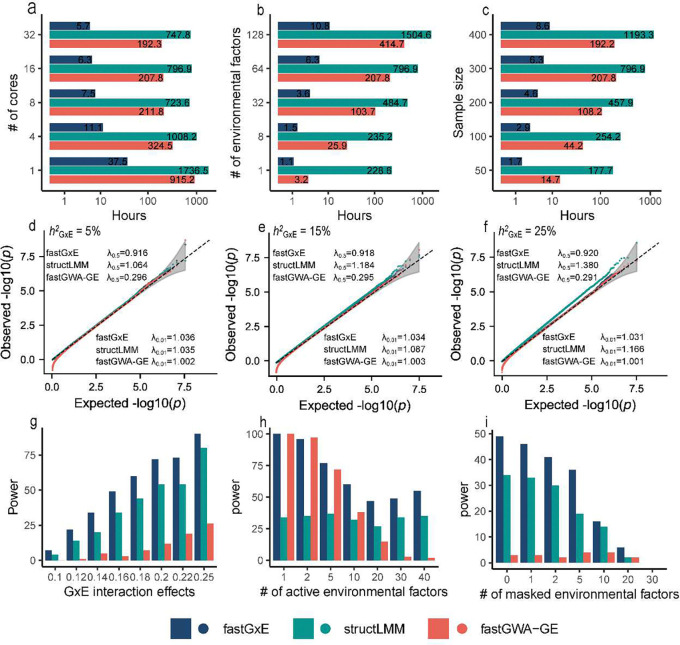
Runtime and simulation comparisons of fastGxE, structLMM, and fastGWA-GE. (a-c) Computing time/runtime across varying numbers of CPU cores, number of environmental factors, and sample sizes for a total of 7,833,003 SNPs. For StructLMM, the computation was performed on 5,000 SNPs, with runtime linearly extrapolated to the full SNP set. All analyses were conducted on a computing platform equipped with dual Intel Xeon Gold 6138 CPUs, each operating at 2.00 GHz and comprising 20 cores and 40 threads; (d-f) Quantile-quantile plots of −log10 *p*-values from SNPs on even-numbered chromosomes without GxE effects, under GxE heritability levels of 5% (d), 15% (e), and 25% (f), simulated using SNPs from odd-numbered chromosomes; (g-i) Power comparisons at varying levels of GxE interaction effects (g), different numbers of active interacting environmental factors (h), or varying numbers of masked active environmental factors – those contributing to GxE interactions but omitted from the analysis.

fastGxE (Figure 1) is described in detail in Methods, with technical specifics provided in **Supplementary Note**. We first assessed fastGxE’s computational efficiency by comparing it to the single-environment GxE method fastGWA-GE and the multi-environment GxE method StructLMM across varying numbers of CPU cores, number of environmental factors, and sample sizes (Figure 2a-c). As the number of CPU cores increased, the runtime for each method decreased, but fastGxE exhibited much greater reduction, demonstrating its superior multithreading performance. Additionally, as the number of environmental factors increased, fastGxE’s runtime grew at a much slower rate compared to the other methods, supporting its better scalability. fastGxE also completed the analyses faster even when applied to a single environmental factor, despite being specifically designed as a multi-environmental GxE method. This computational efficiency makes fastGxE particularly well-suited for large biobank datasets. For example, in an analysis of 300,000 individuals with 7.8 million SNPs across 64 environmental factors, fastGxE completed the analysis in just 6.3 hours using 16 cores—32.98 times faster than fastGWA-GE and 126.49 times faster than StructLMM, making it feasible for conducting genome-wide GxE analysis.

### Simulations

We performed comprehensive simulations to evaluate the performance of fastGxE and compared it with StructLMM, and fastGWA-GE. First, we examined null settings to evaluate their type I error control across varying levels of GxE heritability $\left(h_{GxE}^{2}=5\%,\;15\%,\;25\%\right.)$. Both quantile-quantile (QQ) plots and genomic inflation factors (λ) at different quantiles demonstrated that the type I error from fastGxE were well-controlled (Figure 2d-f and **Supplementary Table 1**). For instance, at the 1% quantile, the genomic inflation factors ($\lambda_{0.01}$) from fastGxE were 1.036, 1.034, and 1.031, for $h_{GxE}^{2}$ values of 5%, 15%, and 25%, respectively; while at the usual 50% quantile, the genomic inflation factors ($\lambda_{0.5}$) were 0.916, 0.918, and 0.920. fastGWA-GE yielded calibrated *p*-values at smaller quantiles but displayed extremely conservative/deflated p-values at higher quantiles: $\lambda_{0.01}$ are 1.002, 1.003, and 1.001, which are reduced to only 0.296, 0.295, and 0.291 at 50% quantile, for the three $h_{GxE}^{2}$ values, respectively. StructLMM yielded reasonably calibrated p-values at small $h_{GxE}^{2}$ but displayed slightly inflated *p*-values as $h_{GxE}^{2}$ increased. For example, when $h_{GxE}^{2}$ increases from 5% to 25%, $\lambda_{0.5}$ from StructLMM increased from 1.064 to 1.38, while $\lambda_{0.01}$ increased from 1.035 to 1.166. The results support effective type I error control of fastGxE. Next, we assessed the type I error control of three methods under different levels of SNP ($h_{SNP}^{2}=5\%,\;30\%,\;50\%)$. Based on QQ plots and genomic inflation factors across various quantiles, fastGxE maintained robust type I error control, while increasing SNP heritability levels had minimal impact on the type I error of StructLMM (**Supplementary Figure 1a-c**).

Then, we compared the power of different methods at a 1% family-wise error rate. In the power analysis, we examined the influence of GxE interaction effect sizes, the number of interacting environmental factors, and the number of masked environmental factors – those contributing to GxE interactions but omitted from the analysis. We found that fastGxE consistently displayed higher power than fastGWA-GE and StructLMM across all scenarios (Figure 2g-i). Specifically, as GxE interaction effect sizes decreased, the power of all methods reduced; however, fastGxE maintained higher power compared to the other two methods (Figure 2g). As the number of interacting environmental factors increased, the power of fastGWA-GE declined rapidly, indicating that single-environment GxE methods struggle to maintain power when SNP interacts with multiple environmental factors. In contrast, the multi-environment methods fastGxE and StructLMM maintained higher power, with fastGxE demonstrating the highest power among methods compared (Figure 2h). In the masked environment settings, as the number of masked environmental factors increased, the power of all methods reduced; however, fastGxE consistently maintained its higher power compared to the other two methods (Figure 2i). Similarly, across varying levels of GxE heritability and SNP heritability, fastGxE maintained its advantage in power over the other methods (**Supplementary Figure 1d-e**). The results support the high power of fastGxE.

Finally, in addition to testing GxE effects, fastGxE produced unbiased estimates of the overall GxE heritability (**Supplementary Figure 2**). Moreover, fastGxE demonstrates improved accuracy in identifying specific environmental factors driving the detected GxE associations compared to fastGWA-GE across various settings (**Supplementary Figure 3**).

### GxE association analysis for 32 physical traits

**Figure 3.**
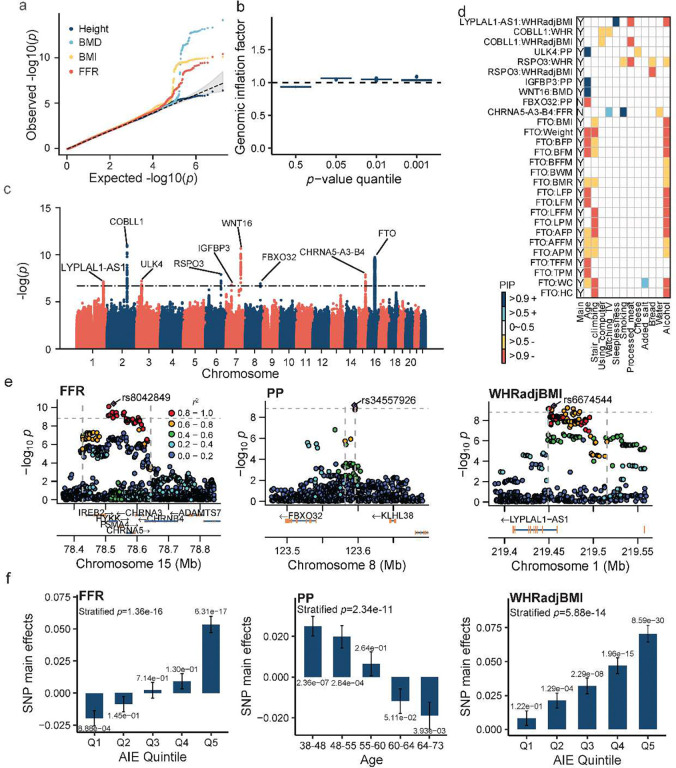
GxE association analysis for 32 physical traits. (a) Examples of QQ plots showing −log_10_ *p*-values for height, heel bone mineral density (BMD), body mass index (BMI), and FEV1/FVC ratio (FFR); (b) Genomic inflation factors at different *p*-value quantiles across 32 traits; (c) Manhattan plot of fastGxE *p*-values across 32 traits. The dotted line represents the significance threshold of 1.56e-9 (5e-8/32); (d) A heatmap displaying the posterior inclusion probability (PIP) for GxE interactions between different environmental factors (x-axis) and the lead SNP in the identified genic locus for various traits (y-axis). The first column shows whether the SNP main effect is significant (*p*-value < 1.56e-09); (c) Locus zoom plot showing −log10 GxE *p*-values for SNPs in the *CHRNA5-A3-B4* locus (15:78,427,159–78,643,826) associated with FFR (left panel), *FBXO32* locus (8:123,581,944–123,596,374) associated with pulse pressure (PP; middle panel), and *LYPLAL1-AS1* locus (1:219,449,254–219,515,292) associated with waist/hip ratio adjusted for BMI (WHRadjBMI; right panel); (f) SNP main effects and corresponding *p*-values in different quintiles of aggregated interacting environment (AIE) score or age for the lead GxE SNP rs8042849 in the *CHRNA5-A3-B4* locus associated with FFR (left panel), rs34557926 in the *FBXO32* locus associated with PP (middle panel), and rs6674544 in the *LYPLAL1-AS1* locus associated with WHRadjBMI (right panel).

We applied fastGxE to conduct a genome-wide GxE analysis of 32 physical traits in 430,145 individuals of white British ancestry from UK Biobank (UKB) (**Supplementary Table 2** and **Supplementary Figure 4**). In the analysis, we used 42 environmental factors (**Supplementary Table 3** and **Supplementary Figure 5**) that include age, Townsend deprivation index, 6 physical activity variables, 4 electronic device usage variables, 6 sleep related variables, smoking status, 11 ordinal dietary intake variables, 9 continuous dietary intake variables, alcohol intake frequency, and 2 social support variables (see Methods and **Supplementary Note** for detail). After excluding individuals with missing phenotype or environmental factor data, the sample sizes analyzed for these traits range from 214,580 to 324,811 (**Supplementary Table 2**). We estimated the proportion of phenotypic variation explained by polygenic effects, also known as SNP heritability, to range from 16.07% to 75.65%, with an average of 29.97%, across traits. Similarly, the estimated proportion of phenotypic variation explained by polygenic GxE effects ranged from 3.33% to 23.27%, with an average of 15.26% across traits (**Supplementary Figure 6**). These estimates highlight the importance of controlling for both polygenic genetic effects and polygenic GxE effects in GxE association analysis. Indeed, by controlling for both polygenic effects and polygenic GxE effects, fastGxE yielded well-calibrated *p*-values across the 32 physical traits, as is evident in both QQ plots (Figure 3a) and genomic inflation factors for different quantiles (Figure 3b and **Supplementary Table 4**). For example, $\lambda_{0.01}$ ranges from 1.022 to 1.070, with an average of 1.046, across traits, and $\lambda_{0.5}$ ranges from 0.911 to 0.950, with an average of 0.933.

Physical traits have been extensively studied in previous GxE research using various methods, with four well-documented significant genomic loci previously identified to interact with age and lifestyle environmental factors (**Supplementary Table 5**, see Methods). In this study, fastGxE identified nine genomic loci containing 28 significant GxE associations across 23 traits (Table 1, Figure 3c), effectively doubling the number of discoveries. Three of these loci have been previously reported with evidence of GxE effects, including: *FTO*, which influences 18 traits, including BMI^6,14^; the *CHRNA5-CHRNA3-CHRNB4* gene cluster, which is associated with FFR^13^; and *WNT16*, which is linked to heel bone mineral density (BMD)^13,18^. Although fastGxE did not identify *APOE* for waist circumference (WC) (fastGxE p-value = 7.82e-4, compared to the previous reported p-value = 8.03e-10). For the three known loci, fastGxE detected additional environmental factors and additional traits in the GxE associations. The remaining six loci represent novel findings, including: *IGFBP3*, *FBXO32*, and *ULK4* for pulse pressure (PP), *LYPLAL1-AS1* for waist/hip ratio adjusted by BMI (WHRadjBMI), *COBLL1* and *RSPO3* for both WHRadjBMI and WHR. Among the nine detected loci, fastGxE identified an average of 2.18 environmental factors (range=1–4) interacting with the significant GxE SNPs, leading to a total of 61 significant SNP-environment interacting pairs across 23 traits (Figure 3d, **Supplementary Table 6**). Of these interacting pairs, 54 exhibited opposite effects compared to the SNP main effects, where increasing levels of the environmental factors reduce the genetic effects, while the remaining seven exhibited consistent effects. The top environmental factors driving the observed GxE signals include alcohol intake frequency (which interacts with 7 SNPs across 18 traits), age (8 SNPs across 14 traits), and stair climbing frequency (5 SNPs across 12 traits) (**Supplementary Figure 7**).

We first visualized and validated the GxE discoveries by dividing individuals into five groups based on their aggregated interacting environment (AIE) scores and performing stratified analysis (see Methods for detail; **Supplementary Table 7**). All 28 GxE signals identified above display significant SNP main effects in at least one group (*p* < 1e-8), with three displaying significant SNP main effects in all five groups. In addition, all 28 GxE signals display significant difference in SNP main effects across groups (*p* < 5e-8), supporting GxE interaction effects. We carefully examined each of the nine genomic loci below.

Next, we focus on the three GxE loci also identified in previously studies but for which fastGxE detected additional environmental factors and additional traits in the GxE associations. The first is the *FTO* locus (16:53,763,996–53,811,575), which is identified by fastGxE to display significant GxE associations with 18 traits, including BMI, weight, WC, hip circumference (HC), basal metabolic rate (BMR), and body fat percentage (BFP) (Table 1). Although the lead interaction SNP varies across traits, these SNPs display high linkage disequilibrium (LD) among them (*r*^2^ > 0.85) (**Supplementary Figure 8**). The key environmental factors driving these GxE interactions also vary across different traits (Figure 3d), with some exhibiting effects opposite to the SNP main effects (alcohol intake frequency, stair climbing frequency, age, usual walking pace, moderate physical activity and vigorous physical activity) and others exhibiting effects that are consistent (processed meat intake and poultry intake). These findings support previous observations that the *FTO* locus interacts with multiple lifestyle factors—such as alcohol intake frequency, age, dietary habits, sedentary time, and physical activities—to influence BMI and WC, with alcohol intake frequency, age, and physical activities mitigating its impact, while unhealthy diets may amplify it^6,14^. Indeed, enhancers of *FTO* display long-range effects on *IRX*3, facilitating the regulation of body's energy use by controlling sympathetic nerves to brown fat tissue^19^.

The second is the *CHRNA5-A3-B4* locus (15:78,427,159–78,643,826), which displays significant GxE association with FFR, with rs8042849 identified as the lead SNP (*p*_GxE_ = 3.98e-11; *p*_main_ = 1.75e-2; Table 1, Figure 3e). The key environmental factors driving the GxE effect include water intake, which mitigates the SNP main effect, and smoking status and time spent watching television, which exacerbate its effect (Figure 3d). AIE score stratified analysis confirmed GxE pattern (*p*-value = 1.36e-16), with individuals in the top 20% AIE score group exhibiting a significant SNP main effect (*p*-value = 6.31e-17), while those in the bottom 20% did not (*p*-value = 8.88e-4) (Figure 3f and **Supplementary Table 7)**. rs8042849 is located in the intronic region of *HYKK*, close to the *CHRNA5-A3-B4* gene cluster. rs8042849 is not an eQTL for *HYKK* but influences the expression of *CHRNA5* and *CHRNA3* in brain tissues (GTEx V8; **Supplementary Table 8**). These findings support previous observations that smoking is a key environmental factor interacting with rs8042849 and rs56077333 (LD *r*^2^=0.86) in the *CHRNA5-A3-B4* gene cluster to influence lung function, specifically the FFR^13,18^. Indeed, SNPs in these genes are associated with variations in reward-seeking behaviors and susceptibility to addiction^20,21^. Consequently, behaviors associated with reward-seeking, such as smoking and time spent watching television, may modify the effect of *CHRNA5-A3-B4* on lung function.

The third is the *WNT16* locus (7:121,270,692–121,393,067), which shows a significant GxE association with BMD, with rs2908007 identified as the lead SNP (*p*_GxE_ = 1.62e-13; *p*_main_ = 0; Table 1, **Supplementary Figure 9a**). Age is a key environment factor driving this GxE signal, with increased age amplifying the effect of the risk allele (Figure 3d). Age-stratified analysis confirmed GxE interaction pattern (*p*-value=7.76e-16), with SNP main effects significant in all five age groups, showing stronger effect with the increasing of age (**Supplementary Figure 9b**). Recent studies have reported that rs6952851 (in an LD *r*^2^=0.59 with rs2908007) and rs10254825 (in an LD *r*^2^=0.99 with rs2908007) influence BMD through genotype-age interactions^13,18^. The nearest protein-coding gene, *WNT16* (Wnt Family Member 16), is a major determinant of cortical bone thickness and nonvertebral fracture risk. Mechanistic studies revealed that osteoblast-derived *WNT16* inhibits osteoclastogenesis both directly by acting on osteoclast progenitors and indirectly by increasing Opg expression in osteoblasts^22^. *WNT16* knockout mice (*WNT16*−/−) exhibit significantly weaker bones and a higher risk of fractures as they age^22^, underscoring the protective role of *WNT16* in maintaining bone integrity and reducing fracture risk, especially in aging mice.

We now shift our focus towards the six novel GxE loci detected in the present study. The first is the *IGFBP3* locus (7:45,921,046–45,970,501), which shows significant GxE association with PP, with rs11977526 identified as the lead SNP (*p*_GxE_ = 1.02e-9; *p*_main_ = 1.51e-38; Table 1, **Supplementary Figure 10a**). rs11977526 has been previously identified to display heterogeneous effect sizes on PP across the three genotypes^23^, which is validated as GxE interaction in the present study. Age is the key environmental factor driving the GxE signal, with increasing age enhancing SNP main effect (Figure 3d). Age-stratified analysis confirmed the significant GxE pattern (*p*-value = 3.50e-13), with SNP main effect significant in individuals over 55 years of age but not under (**Supplementary Figure 10b**). rs11977526 is located upstream of *IGFBP3* and acts as its eQTL in both the left ventricle (*p*-value = 1.91e-7) and aorta artery (*p*-value = 1.26e-5) (GTEx V8; **Supplementary Table 8**). *IGFBP3* encodes insulin-like growth factor binding protein 3 (IGFBP-3), which binds to insulin-like growth factor 1 (IGF-1) to regulate its bioavailability and activity. IGF-1 mediates the regulation of blood pressure by stimulating endothelial nitric oxide synthase activity, which leads to increased production of nitric oxide in endothelial cells, promoting vasodilation and increasing blood flow^24^. A previous study indicates that the IGF-1/IGFBP-3 molar ratio decreases with age^25^. Therefore, the GxE effect of rs11977526 on PP may be attributed to genotype-dependent changes in the binding activity of IGFBP-3 to IGF-1 with respect to age.

The second is the *FBXO32* locus (8:123,581,944–123,596,374), which shows significant GxE association with PP, with rs34557926 identified as the lead SNP (*p*_GxE_ = 7.19e-10; *p*_main_ = 0.195; Table 1, Figure 3e). Age is the key environmental factor driving the GxE signal, with increasing age reducing the SNP main effect (Figure 3d). Age-stratified analysis confirmed the significant GxE effect (*p*-value = 2.34e-11), with SNP effect in the younger group (38–48) opposite to that in the older group (64–73) (Figure 3f). The nearest protein-coding gene to rs34557926, *FBXO32*, belongs to the F-Box protein family and is closely related to muscle atrophy^26,27^ and dilated cardiomyopathy^27^, both of which are influenced by age. This suggests that age may modify *FBXO32*’s regulatory effect on cardiovascular dynamics, potentially impacting PP.

The third is the *ULK4* locus (3:41,708,768–42,023,513), which displays significant GxE association with PP, with rs7647561 identified as the lead SNP (*p*_GxE_ = 3.19e-10; *p*_main_ = 1.59e-44; see Table 1, **Supplementary Figure 11a**). Both age and cheese intake frequency are key environmental factors driving the GxE signal, with increasing age enhancing the SNP effect and increasing cheese intake frequency reducing it (Figure 3d). AIE score-stratified analysis confirmed the significant GxE pattern (*p*-value = 5.04e-12), with SNP effect particularly significant in the older age group with lower cheese intake frequency (**Supplementary Figure 11b-c**). The protein-coding gene *ULK4*, located near rs7647561, is associated with blood pressure regulation and hypertension, though its exact regulatory mechanism remains unknown. We also identified rs7651190 as an eQTL for *ULK4* in artery, left ventricle of heart, and whole blood (GTEx V8; **Supplementary Table 8**). *ULK4* is associated with the age of first onset of type B aortic dissection, with higher *ULK4* expression linked to a later onset^28^. This evidence supports *ULK4*'s involvement in vascular health and suggests that it may play a role in age-related cardiovascular traits.

The last three GxE loci are all associated with WHR and/or WHRadjBMI, a proxy of body fat distribution. Specifically, the *LYPLAL1-AS1* locus (1:219,449,254–219,515,292) displays significant GxE association with WHRadjBMI, with rs6674544 identified as the lead SNP (*p*_GxE_ = 3.81e-10; *p*_main_ = 1.02e-45; Table 1, Figure 3e). Key environmental factors driving such association include alcohol intake frequency, processed meat intake, and sleeplessness (Figure 3d). AIE score-stratified test confirmed the significant GxE pattern (*p*-value = 5.88e-14), with SNP effect on WHRadjBMI significant among the top 20% of individuals with high AIE scores but nonsignificant in the bottom 20% (Figure 3f). rs6674544 is located in the first intron of *LYPLAL1-AS1* and acts as both an eQTL (*p* = 6.2e-5) and sQTL (*p* = 3.2e-39) for this gene in subcutaneous adipose tissue (GTEx V8; **Supplementary Table 8**). *LYPLAL1-AS1*, a poorly studied long non-coding RNA, contains a PPARG binding site located just 13bp downstream of its transcription start site. PPAR-gamma, encoded by *PPARG*, is a key regulator of adipocyte differentiation, likely exerting its effect through the PPARG binding site of *LYPLAL1-AS1* to modulate its gene expression. The sensitivity of PPAR-gamma to dietary fat intake^29^ can result in allele-specific expression of *LYPLAL1-AS1*. This differential expression may contribute to variations in body fat distribution, particularly influencing WHRadjBMI, as observed in individuals with different environmental exposures like alcohol consumption, processed meat intake, and sleep patterns.

The *COBLL1* locus (2:165,501,849–165,580,775) contains significant GxE association with both WHRadjBMI and WHR, with rs13389219 identified as the lead SNP (WHRadjBMI *p*_GxE_ = 2.83e-15; *p*_main_ = 3.18e-48; WHR *p*_GxE_ = 1.73e-12; *p*_main_ = 1.54e-29) (Table 1, **Supplementary Figure 12**). Key environmental factors driving the GxE signals include the processed meat intake and time spent using computer for WHRadjBMI, as well as time spent using computer and time spent watching television for WHR (Figure 3d). rs13389219 is an intron variant of *COBLL1* and acts as its sQTL in visceral adipose (Omentum) (*p*=3.5e-12) and the subcutaneous adipose tissue (*p*=1.9e-8) (GTEx V8; **Supplementary Table 8**). Its nearby SNP, rs6712203 (LD *r*^2^ = 0.73 with rs13389219), influences the enhancer activity of *COBLL1* in subcutaneous adipocytes and subsequently its gene expression^30^. Perturbations of *COBLL1* in a mouse model leads to reduced subcutaneous fat mass, lower body weight, and impaired glucose tolerance^30^. A low-fat diet displays protective effects against the influence of rs6717858 (LD *r*^2^ = 0.95 with rs13389219) within *COBLL1* on obesity risk^31^. The presence of specific alleles at rs13389219, combined with an individual’s lifestyle, likely influences *COBLL1* activity, contributing to variations in fat distribution and overall metabolic health.

The *RSPO3* locus (6:127,118,752–127,208,635) contains significant GxE association with both WHRadjBMI and WHR, with rs577721086 identified as the lead SNPs for WHRadjBMI (*p*_GxE_ = 3.73E-11; *p*_main_ = 4.39E-188) and rs72961013 for WHR (*p*_GxE_ = 3.80E-10; *p*_main_ = 5.10E-108) (LD *r*^2^ = 0.718 between rs577721086 and rs72961013) (Table 1, **Supplementary Figure 13**). Key environmental factors driving the GxE signals include bread intake for WHRadjBMI, and bread intake, alcohol intake, processed meat intake, and smoking for WHR (Figure 3d). The variant rs577721086 is located within the promoter and 5’UTR of the *RSPO3* gene, whereas rs72961013 is situated 9kb downstream of *RSPO3*. Both rs577721086 and rs72961013 are eQTL for *RSPO3* in subcutaneous adipose (*p*-value=1.22e-6; *p*-value=1.48e-4) (GTEx V8; **Supplementary Table 8**). *RSPO3* contains PPARG binding site in adipose-derived stem cells. *RSPO3* limits gluteofemoral adipose tissue expansion by suppressing adipogenesis and increasing gluteal adipocyte apoptosis. *RSPO3* also promotes upper-body fat distribution by stimulating abdominal adipose progenitor proliferation. These distinct effects in abdominal and gluteal adipose progenitors are linked to differential WNT signaling responses^32^. *RSPO3*'s involvement in modulating adipose tissue dynamics via the WNT signaling pathway may contribute to gene-environment interplay, potentially affecting body fat distribution patterns and susceptibility to metabolic diseases like obesity.

### GxE association analysis for 67 blood biomarkers

**Figure 4.**
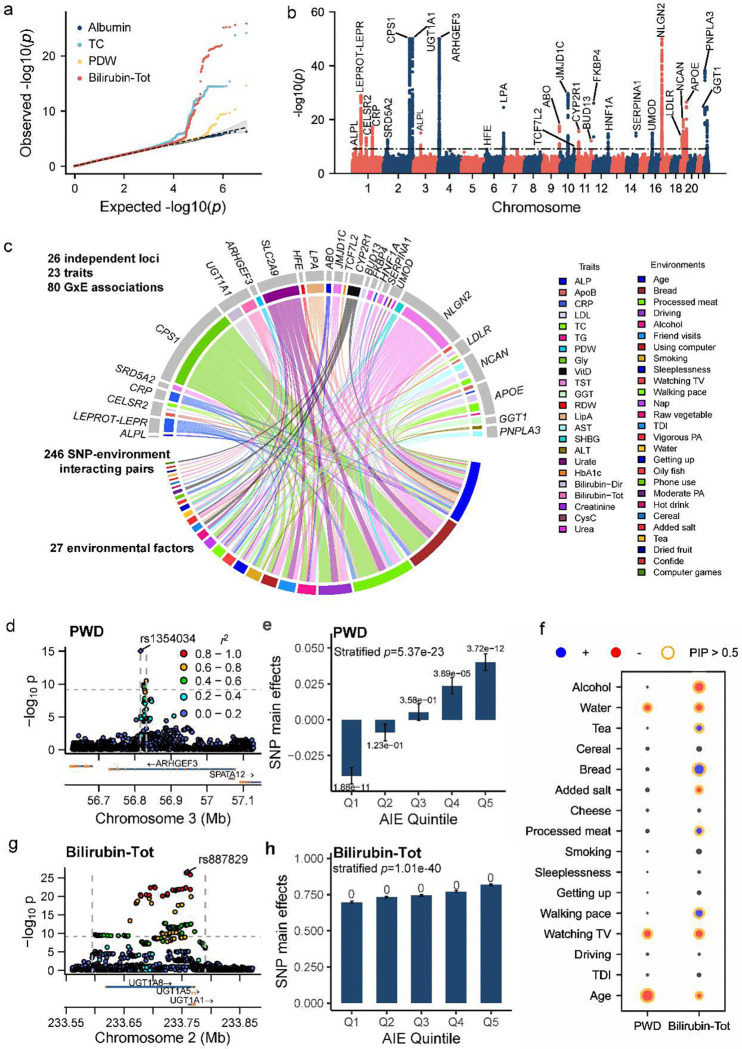
GxE association analysis for 67 blood biomarkers. (a) Examples of QQ plots showing −log10 p-values for albumin, total cholesterol (TC), platelet distribution width (PWD), and total bilirubin (Bilirubin-Tot); (c) Manhattan plot of fastGxE *p*-values across 67 traits. The dotted line represents the significance threshold of 7.46e-10 (5e-8/67); (c) A chord diagram illustrating detected GxE associations in a specific genic locus (first layer, top circle), for a given trait (second layer, top circle), and interacting with specific environment factors (bottom circle); (d) Locus zoom plot showing −log10 GxE *p*-values for SNPs in the *ARHGEF3* locus (3:56,815,721–56,831,748) associated with platelet distribution width (PWD); (e) Estimated SNP main effects with corresponding *p*-values across quintiles of the aggregated interacting environment (AIE) scores for the lead GxE SNP rs1354034 in the *ARHGEF3* locus associated with PWD; (f) For each environmental factor (y-axis), the GxE effect size estimates (different dot sizes) and the effect signs (blue: same as the SNP main effect; red: opposite) are shown for the lead GxE SNP in the *ARHGEF3* locus associated with PWD (left) and the lead GxE SNP in the *UGT1A1* locus associated with total bilirubin (right). Instances with PIPs greater than 0.5 are highlighted with an orange circle; (g) Locus zoom plot showing −log10 GxE *p*-values for SNPs in the *UGT1A1* locus (2:233,595,452–233,790,330) associated with Bilirubin-Tot; (h) Estimated SNP main effects with corresponding *p*-values across quintiles of AIE scores for the lead GxE SNP rs887829 in the *UGT1A1* locus associated with total bilirubin.

We further applied fastGxE to conduct a genome-wide GxE analysis of 67 blood biomarkers in UKB. After excluding individuals with missing phenotype or environmental factor data, the sample sizes analyzed for these traits ranged from 178,899 to 312,887 (**Supplementary Table 9, Supplementary Figure 14**). We estimated the SNP heritability to range from 4.44% to 69.36%, with an average of 29.83% across traits. Similarly, the estimated proportion of phenotypic variation explained by polygenic GxE effects ranged from 0% to 20.13%, with an average of 6.99% across traits **(Supplementary Figure 15**). Consistent with simulations and the analysis of the 32 physical traits, fastGxE also yielded well-calibrated *p*-values across the 67 blood biomarkers, as is evident by both QQ plots (Figure 4a) and genomic inflation factors for different quantiles (**Supplementary Figure 16** and **Supplementary Table 10**). For example, $\lambda_{0.01}$ ranges from 1.016 to 1.048, with an average of 1.029, across traits, and $\lambda_{0.5}$ ranges from 0.885 to 0.924, with an average of 0.911.

In the analysis, fastGxE identified a total of 26 genomic loci containing 80 significant GxE associations across 23 traits (Table 2, Figure 4b-c). The identified loci substantially expanded the 11 previously known GxE loci associated with blood biomarkers, effectively doubling the number of discoveries (**Supplementary Table 11**). Among the identified 26 loci, seven loci had been previously reported with GxE evidence, while the remaining 19 represent novel discoveries. For the seven previously reported loci, fastGxE also identified additional environmental factors and traits associated with GxE effects. Across the 26 loci, fastGxE identified an average of 3.075 environmental factors (range=1–9) interacting with the significant GxE SNPs, resulting in a total of 246 SNP-environment interacting pairs across the 23 traits (Figure 4a, **Supplementary Figure 17, Supplementary Table 12**). Of these interacting pairs, 145 exhibited opposite effects compared to the SNP main effects, where increasing levels of the environmental factors reduce the genetic effects, while the remaining 101 exhibited consistent effects. The top environmental factors driving the observed GxE signals include age (which interacts with 32 SNPs for 23 traits), processed meat intake (40 SNPs for 10 traits), and bread intake (37 SNPs for 10 traits) (**Supplementary Figure 18**).

We visualized and validated the GxE findings through an AIE-stratified analysis (**Supplementary Table 13**). All 80 GxE signals identified above exhibit significant SNP main effects in at least one group. Of these, 60 signals show significant effects across all five groups, while the remaining 20 demonstrate significant effects in specific groups. Notably, three signals display reverse effect sizes between the two extreme groups but have significant SNP main effects in only one group. Furthermore, all 80 GxE signals exhibit significant differences in SNP main effects across groups (*p* < 5e-8), supporting GxE interaction effects.

We list three example genomic loci identified by fastGxE. The *APOE* locus (19:44,728,895–44,923,868) exhibits significant GxE associations with five traits: total cholesterol (TC), triglycerides (TG), low-density lipoprotein (LDL), apolipoprotein B (ApoB), and C-reactive protein (CRP). This locus overlaps with the *APOE*/*APOC* gene cluster and shows strong evidence of SNP main effects across all five traits (Table 2). A previous study^33^ identified rs5117 in the locus to display heterogeneous effects on nine biomarkers—including lipids (TC, LDL, HDL, TG), lipoproteins (ApoA, ApoB, LipA), liver enzymes (ALT), and hsCRP—across three genotype groups. In our analysis, we identified two independent lead SNPs, rs429358 (T > C; LD *r*^2^ = 0.57 with rs5117) and rs7412 (C > T; LD *r*^2^ = 0.28 with rs5117), which exhibit low LD (LD *r*^2^ = 0.016) with each other. The two SNPs showed significant GxE interactions for TC, LDL, ApoB, and CRP. Additionally, we identified a third independent SNP, rs769449 (LD *r*^2^ = 0.78 with rs429358 and LD *r*^2^ = 0.012 with rs7412), which demonstrated significant GxE interactions with TG. rs429358 and rs7412 form three alleles: ε2 (rs429358T-rs7412T), ε3 (rs429358T-rs7412C), and ε4 (rs429358C-rs7412C), which encode the three major isoforms of *APOE*—E2, E3, and E4^34^. *APOE* plays a central role in cholesterol metabolism and transport, with the ɛ4 allele associated with higher total cholesterol and LDL^35^. We identified that age, bread intake, smoking and watching television time as key drivers behind the observed GxE interactions, with all these factors reducing SNP main effects (**Supplementary Figure 17**). Previous studies suggested that while ε4 allele is a significant cardiovascular risk factor in early middle age, other risk factors become more important role at older age^36^. The ε4 allele affects sensitivity to dietary interventions^37,38^, with ε4 carriers showing increased total cholesterol and LDL levels on high-fat, high-cholesterol diet^39^. In addition, smoking have more negative effects on lipids in ε4 carriers, likely due to increased oxidative stress, which exacerbates lipid metabolism abnormalities in these individuals^40^. Therefore, the SNPs identified in the present study likely interact with various environmental factors to influence biomarker traits.

The *ARHGEF3* locus (3:56,815,721–56,831,748) contains SNPs with significant GxE associations with platelet distribution width (PWD), with the lead SNP being rs1354034 (*p*_GxE_=9.44e-16, *p*_main_=8.86e-2) (Table 2, 4b-c,f). The AIE-stratified analysis confirmed a significant GxE pattern (*p*-value = 5.37e-23), with the SNP main effect being significant in individuals within the top 20% and bottom 20% of the AIE group, while exhibiting opposite effect directions. rs1354034 was previously identified to influence pulse pressure through a genotype-age interaction (*p*-value = 9.09e-16)^41^. Our study further uncovered two additional lifestyle factors—time spent watching television and water intake—that may also contribute to the GxE interaction signal for PDW. Notably, all identified environmental factors attenuate the SNP main effects. PWD reflects the variability in platelet size, with higher values indicating greater variation and an association with platelet activation^42^. SNP rs1354034 has been linked to the platelet-specific expression of *ARHGEF3*^43,44^. The *ARHGEF3* gene encodes Rho Guanine Nucleotide Exchange Factor 3 and is significantly upregulated during the maturation of human megakaryocytes. Notably, rs1354034 resides in an open chromatin region and may disrupt protein-DNA interactions in megakaryocytes, potentially perturbing gene function^44^. Environmental factors can further influence the state of open chromatin regions, thereby modulating allele-specific expression^45^.

The *UGT1A1* locus (2:233,595,452–233,790,330) contains SNPs with significant GxE associations with total bilirubin and direct bilirubin, with rs887829 identified as the lead SNP (total bilirubin *p*_GxE_ = 2.55e-27; *p*_main_ = 0; direct bilirubin *p*_GxE_ = 3.11e-56; *p*_main_ = 0; see Table 2, Figure 4d-f). A total of 13 environmental factors contributed to the identified GxE signals for total bilirubin and/or direct bilirubin. Among these, age, TDI, time spent watching television, sleeplessness, smoking status, water intake, salt added to food, and alcohol intake frequency were found to reduce the SNP main effect, while usual walking pace, processed meat intake, bread intake, cereal intake, and tea intake were observed to enhance it. The AIE-stratified analysis confirmed a significant GxE pattern (total bilirubin *p*-value = 1.01e-40; direct bilirubin *p*-value = 6.82e-73), with the SNP main effect being significant in all five AIE groups. We also identified rs887829 as an eQTL for *UGT1A1* in liver (*p*-value = 8.7e-10; GTEx V8). *UGT1A1* encodes UDP-glucuronosyltransferase, a key enzyme involved in the glucuronidation of bilirubin, facilitating its excretion and metabolic homeostasis. Lifestyle factors such as diet, alcohol consumption, smoking, and sleep patterns influence *UGT1A1* activity, thereby altering its detoxification capacity and subsequently bilirubin metabolism^46–48^.

## Discussion

We have presented fastGxE, a scalable and effective method for genome-wide GxE analysis to detect SNPs that interact with one or multiple environmental factors to influence traits of interest in large biobank studies. Compared to traditional GxE methods, fastGxE offers several key advantages: it accounts for both polygenic effects and polygenic interaction effects, providing calibrated type I error control; it maintains robustness and high statistical power regardless of the number of environmental factors involved in GxE interactions; and it ensures high scalability for genome-wide analyses. We have demonstrated the advantages of fastGxE’s through comprehensive simulations and in-depth analysis of 32 physical traits and 67 blood biomarkers in UKB.

fastGxE is not without limitations. First, we have primarily focused on analyzing quantitative traits using fastGxE. For case-control studies, we can follow standard approaches to treat binary phenotypes as continuous outcomes, which is justified by recognizing the linear model as a first-order Taylor approximation to a generalized linear model^49^. However, extending fastGxE in the future to directly accommodate case-control data or other discrete data types in a principled manner would be desirable. One potential approach could involve integrating fastGxE into the generalized linear model framework, allowing for a more accurate and robust analysis of these discrete phenotypes. Second, similar to marginal GWAS association analysis, SNPs identified by fastGxE may tag causal SNPs due to LD without necessarily being causal themselves. Therefore, extending fastGxE to fine-mapping GxE interactions within the framework of GWAS fine-mapping^50,51^ would improve its ability to detect potentially causal SNPs driving GxE interactions. Such an extension, however, is unlikely to be straightforward, as it would require modeling the dependence between marginal and GxE interaction effects^52^ while also ensuring computational scalability. Lastly, fastGxE is limited to analyzing one genetic ancestry at a time. Extending fastGxE to accommodate multiple genetic ancestries would allow for the borrowing of information across ancestries, potentially increasing statistical power and enabling more effective and robust GxE analyses in the future.

## Methods

### Fast and Scalable Multivariate Genotype-environment Interactions Model

We consider a GWAS that measure an outcome trait of interest on n individuals. This GWAS also measures a set of q different environmental factors for the same individuals. Our goal is to conduct scalable genome-wide GxE analysis to systematically identify SNPs that interact with either all or a subset of these q environmental factors to influence the outcome trait. To do so, we denote y as an n-vector of phenotypic measurements, and $X_{E}$ as an n by q matrix of environmental factors with $x_{E}^{\left(k\right)}$ being the kth column representing the kth measured environmental factor (k∈{1,⋯,q}). We examine one SNP at a time and denote $x_{G}$ as an n-vector of genotypes for the SNP of focus. We standardize the phenotype, each genotype, and each environment factor to have a mean of zero and a standard deviation of one. For the SNP of focus, we consider the following model:

(1) $$y=x_{G}\beta_{G}+\sum_{k=1}^{q}x_{E}^{\left(k\right)}\beta_{E}^{\left(k\right)}+\sum_{k=1}^{q}(x_{G}⊙x_{E}^{\left(k\right)})\beta_{GxE}^{\left(k\right)}+g+v+e$$

where $\beta_{G}$ is the genetic main effect size; $\beta_{E}^{\left(k\right)}$ is the main effect size of k-th environmental factor; $\beta_{GxE}^{\left(k\right)}$ is the interaction effect between the SNP and k-th environmental factor; ⊙ denotes the Hadamard product, representing element-wise multiplication between two vectors; g is a n-vector of random effects, capturing polygenetic effects and/or population stratification; v is a n-vector of random effects, capturing the polygenetic GxE effects; and e is a n-vector of residual errors with each element following a normal distribution $N\left(0,\sigma_{e}^{2}\right)$ with mean zero and error variance $\sigma_{e}^{2}$. For g, we assume that it follows a multivariate normal distribution $MVN\left(0,K_{G}\sigma_{g}^{2}\right)$, where $\sigma_{g}^{2}$ represents the genetic variance and $K_{G}$ is the genomic relationship matrix (GRM) calculated using m genome-wide SNPs in the form of $K_{G}=X_{G}X_{G}^{T}/m$ with $X_{G}$ being the n by m genotype matrix. For v, we assume that it follows another multivariate normal distribution, $MVN\left(0,K_{G\times E}\sigma_{v}^{2}\right)$, where $\sigma_{v}^{2}$ represents the interaction variance component and $K_{G\times E}$ is the relationship matrix calculated in the form of $K_{G\times E}=K_{G}⊙K_{E}$, with $K_{E}=X_{E}X_{E}^{T}/q$ serving as the covariance measuring the similarity among individuals in terms of their environmental exposures. The detailed derivation of $K_{G\times E}$ is provided in the **Supplementary Note**.

Our goal is to identify SNPs that interact with either all or a subset of these q environmental factors to influence the outcome trait through testing the null hypothesis $H_{0}:\beta_{GxE}^{\left(k\right)}=0\;\forall k$. This hypothesis could be directly tested using an F test, with degrees of freedom set to be the number of environmental factors. Unfortunately, in the presence of a large number of environmental factors, such F test is known to yield deflated p-values and provide low statistical power^14^. In addition, in the presence of a large number of environmental factors, the statistical power of any test will inevitably depend on the number of environmental factors that the SNP truly interacts with, which is certainly unknown in any real data applications. Therefore, instead of using the F test, we explore a range of tests based on distinct modeling assumptions on the interaction effects $\beta_{GxE}^{\left(k\right)}$, which are further aggregated into a single test statistic.

Specifically, we first consider a set of distinct sparse modeling assumptions where only one $\beta_{GxE}^{\left(k\right)}$ is non-zero. Under each such modeling assumption, the null hypothesis $H_{0}:\beta_{GxE}^{\left(k\right)}=0\;\forall k$ becomes equivalent to the null hypothesis $H_{0}:\beta_{GxE}^{\left(k\right)}=0$ for the specific k. Consequently, for each modeling assumption, we carry out a score test and compute the corresponding p-value. Besides these sparse assumptions, we also consider a polygenic modeling assumption^14^ that each $\beta_{GxE}^{\left(k\right)}$ follows a normal distribution $N(0,\sigma_{G\times E}^{2})$. Under such modeling assumption, the null hypothesis $H_{0}:\beta_{GxE}^{\left(k\right)}=0\;\forall k$ becomes equivalent to the null hypothesis $H_{0}:\sigma_{GxE}^{2}=0$. Consequently, we carry out a variance component test to obtain the p-value under such polygenic assumption.

Because the power of each test depends on whether the modeling assumption fits the unknown truth, we aggregate the p-values from the q single environment GxE tests $\left(p_{k}\right)$ and the variance component test $\left(p_{v}\right)$ into a single p-value using the Cauchy combination rule to achieve robust performance across various scenarios. The combined test statistics is in the form of

(2) $$T=\frac{1}{2}\sum_{k=1}^{q}\frac{1}{q}\tan\left\{\left(0.5-p_{k}\right)\pi \right\}+\frac{1}{2}\tan\left\{\left(0.5-p_{v}\right)\pi \right\},$$

where $p_{k}$ represents the p-value from the corresponding single environment GxE test $H_{0}:\beta_{GxE}^{\left(k\right)}=0;p_{v}$ represents the p-value from the variance component test under the polygenic assumption; and $tan\left\{\left(0.5-p_{k}\right)\pi \right\}$ and $tan\left\{\left(0.5-p_{v}\right)\pi \right\}$ follows a standard Cauchy distribution given that $p_{k}s$ and $p_{v}$ are uniformly distributed under the null. The p-value based on the standard Cauchy distribution is calculated in the form of p≈0.5-arctan(T)/π. The Cauchy rule takes advantage of the fact that combination of Cauchy random variables also follows a Cauchy distribution regardless of whether these random variables are correlated or not. Therefore, the Cauchy combination rule allows us to combine multiple potentially correlated p-values into a single p-value without loss of type I error control.

In the process, our model effectively controls for the polygenic effects through the random effects term g as well as the polygenic GxE effects through the random effects term v. Controlling for polygenic effects is a well-established practice, routinely incorporated into standard genome-wide association studies^16,53^. Accounting for polygenic GxE interaction effects has also becoming increasingly recognized, as recent studies have demonstrated that these interactions can explain a significant proportion of phenotypic variance and should be considered when testing for GxE effects^8,13,54^. Indeed, as demonstrated here, across 32 physical traits and 67 blood biomarkers, the polygenic GxE interaction effects account for up to 23.27% of phenotypic variance, which is substantial, even in comparison to the polygenic effects, which account for up to 75.65% of phenotypic variance.

The above models are computationally challenging to fit for genome-wide analysis, especially in large-scale biobank data. Here, we develop a scalable inference algorithm, relying on multiple computational techniques (details in **Supplementary Note**). First, we use a sparse GRM $K_{G}$ to enhance computational efficiency while preserving accuracy^55–57^. Second, for SNPs that do not display significant main effects (main effect *p*-value > 1.0e-3), we directly set the marginal effect $\beta_{G}=0$ to simplify calculation. Third, we apply GRAMMAR-GAMMA approximation to further improve computational efficiency. Thanks to these three techniques, our method is orders of magnitude faster than fastGWA-GE and StructLMM, enabling genome-wide scans for interaction between genotype and multiple environmental factors at biobank scale, while properly controlling for polygenic confounding that are widely present in association studies. We refer to our method as fast genotype-environment interaction analysis, which is implemented in the fastGxE software freely available at https://xiangzhou.github.io/software/.

### Gene-environment interaction heritability estimation with method of moments

We use the same model in equation (1) without the $x_{G}\beta_{G}$ term to estimate the proportion of phenotypic variance explained by the polygenic gene-environment interaction term, defined as

(3) $$h_{GxE}^{2}=\frac{\sigma_{v}^{2}}{\sigma_{g}^{2}\;+\;\sigma_{v}^{2}\;+\;\sigma_{e}^{2}}$$

For scalable estimation, we developed a method-of-moments (MoM) algorithm based on the MQS framework ^58^. The MoM estimator is derived based on the following equations:

(4) $$\left[\begin{array}{ccc} tr\left(K_{G}K_{G}\right) & tr\left(K_{G}K_{GxE}\right) & tr\left(K_{G}\right)\\ tr\left(K_{GxE}K_{G}\right) & tr\left(K_{GxE}K_{GxE}\right) & tr\left(K_{GxE}\right)\\ tr\left(K_{G}\right) & tr\left(K_{GxE}\right) & tr\left(I_{n}\right) \end{array}\right]\left[\begin{array}{c} {\overset{ˆ}{\sigma }}_{g}^{2}\\ {\overset{ˆ}{\sigma }}_{v}^{2}\\ {\overset{ˆ}{\sigma }}_{e}^{2} \end{array}\right]=\left[\begin{array}{c} y^{T}K_{G}y\\ y^{T}K_{GxE}y\\ y^{T}y \end{array}\right].$$

Estimating the variance components using the equation above poses substantial computational challenges due to the need to evaluate the trace of the product of two matrices. To overcome this computational hurdle, instead of calculating the exact trace, we use a randomized estimator for such trace term based on the formula $tr(KK)=E\left(w^{T}KKw\right)$, where each element in the n-vector w is drawn from a standard normal distribution. In practice, we randomly generate a random n-vector w, compute $w^{T}KKw$, and repeat this process s times. The average value across these replicates then serves as an estimate for tr(KK). We set s=30 in the present study following ^59^. This approach reduces the time complexity for inference from $O\left(n^{2}mq\right)$ to O(nmqs) (details in **Supplementary Note**).

### Detecting environmental factors that drive the observed interaction signal

The significant SNPs identified by fastGxE interact with either all or a subset of environmental factors to influence the outcome trait. However, for any such significant SNP, the specific subset of environmental factors it interacts with remain unknown. To address this, we also developed mmSuSiE implemented within fastGxE, an extension of the recent SuSiE algorithm^50^ specifically designed for mixed-model analysis, to identify the environmental factors driving the detected GxE interactions. Specifically, for each significant GxE SNP detected by fastGxE, we first fitted a linear regression to remove the marginal SNP effect from the trait to obtain trait residual $\tilde{y}$. We then create a set of GxE terms, one for each environmental factor, represented as an n by q matrix $X_{GxE}$. Afterwards, we consider the following model:

(5) $$\tilde{y}=X_{GxE}b_{GxE}+g+v+e$$

with g and v denoting the polygenic effects and polygenic interaction effects obtained from genome-wide SNPs as defined in the previous section, and $b_{GxE}$ denoting the q-vector of corresponding GxE effect sizes for the SNP of focus. We extend the SuSiE algorithm, which does not account for either g or v, to fit the above model, with technical details provided in the **Supplementary Note**. Such extension allows us to obtain the posterior inclusion probabilities (PIPs) for each environment, which represents its contribution to the GxE effect. In this study, we use PIP > 0.5 as the threshold to declare environmental factors that significantly contribute to the interaction effect.

### Environment stratified analysis

We performed stratified analysis based on environment factors to validate and visualize the detected GxE interaction effects. Specifically, we calculated an aggregated interacting environment (AIE) score for each individual, represented as $X_{E}{\hat{\beta }}_{G\times E}$, where ${\hat{\beta }}_{G\times E}$ is estimated from fastGxE-Select. Based on their AIE scores, we divided individuals evenly into five groups. For each group i(i∈{1,2,3,4,5}) in turn, we fitted a linear mixed model to obtain the SNP main effect size estimate ${\overset{ˆ}{\beta }}_{i}$ and its standard error $se({\overset{ˆ}{\beta }}_{i})$, adjusting for gender, age, age^2^, age^3^, gender × age, gender×age^2^, gender×age^3^, a binary indicator for the genotype chip, and the top 20 genetic principal components, but without including a GxE interaction term. We used a significance threshold of 1.0e-8, adjusted from 5e-8 to account for the five groups, to determine the significance of the SNP main effect in each group. In addition, we carried out a Wald test to test the null hypothesis whether the SNP main effects differed between at least two groups, with $H_{0}:\beta_{1}=\beta_{2}=\beta_{3}=\beta_{4}=\beta_{5}$. The null hypothesis can be re-expressed as $H_{0}:R\beta =0\;$, where $\beta ={\left(\beta_{1},\beta_{2},\beta_{3},\beta_{4},\beta_{5}\right)}^{T}\;$and $R=\left[\begin{array}{ccccc} 1 & -1 & 0 & 0 & 0\\ 0 & 1 & -1 & 0 & 0\\ 0 & 0 & 1 & -1 & 0\\ 0 & 0 & 0 & 1 & -1 \end{array}\right]$. The Wald test statistic is constructed as $W=(R\hat{\beta }{)}^{\prime }{\left[Rvar(\hat{\beta })R^{\prime }\right]}^{-1}(R\hat{\beta })$, which follows $\chi^{2}(4)$ under the null. Significance in this test (*p* < 5e-8) would indicate that the SNP effect sizes differ between at least two groups, thus supporting the presence of GxE interaction.

### Compared methods

We compared fastGxE with two existing methods for detecting GxE: fastGWA-GE, implemented in GCTA-1.94.1^13^, and StructLMM, implemented in limix-2.0.6^14^. We did not include PLINK^12^ and GEM^15^ in our comparison for the following reasons. Specifically, PLINK performs single-environment GxE testing using a simple regression model, which has been shown to produce inflated *p*-values^13^. GEM implements a multi-environment GxE test using fixed-effect model with degrees of freedom equal to the number of environments. However, such test is not always well-calibrated, particularly when applied to a large number of environments, and it also exhibits reduced power performance under such conditions^14^. Therefore, we focus our comparison with fastGWA-GE and StructLMM.

fastGWA-GE examines the interaction between a SNP and one environmental factor using linear mixed model (LMM) with a two-step process. In the first step, it fits an LMM, accounting for both polygenic main effects and polygenic interaction effects, to obtain phenotype residuals. In the second step, it treats the phenotype residuals as the outcome and uses a linear regression model for genome-wide analysis to test for GxE effects. In the presence of multiple environmental factors, we applied fastGWA-GE to each environmental factor separately, obtained the minimum p value across all environmental factors to serve as the evidence for SNP interacting with at least one environmental factor, and applied multiple testing correction based on the number of environmental factors examined. StructLMM, on the other hand, employs a variance component test to evaluate the significance of interactions between a SNP and multiple environmental factors. StructLMM does not account for polygenic main effects and polygenic interaction effects, limiting its applicability to populations without stratification or genetic relatedness. To apply StructLMM, we first calculated the genomic relationship matrix among pairs of individuals using genome-wide SNPs. For pairs of individuals with a genetic relatedness greater than 0.05, we excluded one individual from the analysis. In contrast, for fastGWA-GE and fastGxE, we retained all individuals in the analysis.

### Application to UK biobank

The UK Biobank (UKB) is a large-scale biomedical database and research resource containing comprehensive genetic and phenotypic information from half a million UK participants^60^. In this study, we focused on 430,145 individuals of white British ancestry. The genetic data in UKB were collected using two arrays, the UKB Axiom Array and the UK BiLEVE Axiom Array, which are further imputed by the UKB consortium. We used the genotyped data for simulations (details in the simulations section) due to computational reasons and used the imputed data (Version 3) for real data analyses. Here, we performed quality control on the imputed genotype data and retained for analysis 7,833,003 SNPs that met the following criteria: a minor allele frequency (MAF) of at least 0.01, a Hardy-Weinberg equilibrium test with a *p*-value of 1.0e-6 or higher, a genotyping rate above 95%, and an imputation information score of 0.8 or higher. For fastGWA-GE and fastGxE, we included all 430,145 individuals. For StructLMM, we excluded one individual from each pair with a genetic relatedness value greater than 0.05, resulting in a sample size of 331,402.

We analyzed 32 physical traits and 67 blood biomarkers (**Supplementary Table 2**). For each trait in turn, we first conducted a linear regression to adjust for gender, age^2^, age^3^, gender×age, gender×age^2^, gender×age^3^, a binary indicator for the genotype chip, and the top 20 genetic principal components. We obtained trait residuals from the regression and converted them to a standard normal distribution using the rank-based inverse-normal transformation. These transformed values are used as the phenotypes for all analyses. In addition to the outcome traits, we obtained 42 environmental factors with <5% missing data in the genotyped samples. These 42 environment covariates include age, Townsend deprivation index (TDI), 6 physical activity variables, 4 electronic device usage variables, 6 sleep related variables, smoking status, 11 ordinal dietary intake variables, 9 continuous dietary intake variables, alcohol intake frequency, and 2 social support variables (**Supplementary Table 3** and **Supplementary Note**).

We applied Bonferroni correction to account for the multiple testing incurred during the analysis of 32 physical traits and 67 blood biomarkers. The significance thresholds were set at 1.56e-9 for physical traits and 7.46e-10 for blood biomarkers, corresponding to 5e-8 divided by 32 and 67, respectively. For the detected significant associations, we further defined genomic risk loci using FUMA^61^. FUMA first selects GxE SNPs that meet a preset threshold and ranks them by *p*-value in ascending order. It then examines SNPs one at a time, performing LD clumping for the SNP of focus to exclude SNPs that are in high LD (LD *r*^2^ > 0.6). This process is repeated until all selected variants have LD *r*^2^ < 0.6 relative to the previously selected variants, ensuring only independent significant variants are retained. FUMA proceeded to construct LD blocks based on these selected independent significant variants, by tagging all variants that exhibited LD (*r*^2^ ≥ 0.6) with at least one of the independent significant variants. Building on these selected independent significant variants, FUMA also identified the independent lead variants, which were characterized as having the smallest *p*-value while being independent from each other (*r*^2^ < 0.1). When LD blocks of independent significant variants were closely positioned (<250 kb based on the closest boundary variants of LD blocks), they were combined into a single genomic locus. Therefore, each genomic locus could include multiple independent significant variants. This approach was applied to each trait to create trait-specific loci. Loci associated with various traits were combined when they showed overlapping areas.

We examined previous GxE association studies for physical traits and blood biomarkers^6,14,18,33,41^. The environmental factors considered in these previous studies were age and lifestyle. We defined previous GxE associations as the SNPs with GxE *p*-values below 5e-8 (see **Supplementary Tables 5** and **Supplementary Tables 10**). The fastGxE detected genomic loci that included these previous GxE associations were referred to as known discoveries, while those without were considered novel findings.

### Running time comparison

We compared the computational efficiency of fastGxE with StructLMM and fastGWA-GE in the UKB. Because StructLMM is not scalable to genome-wide analysis in real datasets, we carried out analysis on 5,000 SNPs and linearly projected its running time for larger number of SNPs. The running times of these methods were recorded under different numbers of CPU cores (1, 4, 8, 16, and 32), environmental factors (1, 8, 16, 32, 64, and 128), and samples (50,000; 100,000; 200,000; 300,000; and 400,000). The comparison was conducted on a computing platform equipped with dual Intel Xeon Gold 6138 CPUs, each operating at 2.00 GHz and comprising a total of 20 cores and 40 threads.

### Simulations

Following previous studies^56^, we used real genotype data from UKB to simulate phenotypes. We focused on genotyped data for computational reasons and randomly selected 50,000 individuals from UKB. After filtering SNPs with a minor allele frequency (MAF) of less than 0.01, a Hardy-Weinberg equilibrium test *p*-value of 1.0e-6 or lower, and a missing genotyping rate above 5%, 329,256 SNPs remained. We used the same 40 environmental factors described above, excluding frequency of friend/family visits and able to confide, in the simulations. We simulated the marginal environmental effects of each environmental factor from a normal distribution and scaled their effect sizes so that in total the marginal environmental effects account for 5% of phenotypic variance. To simulate polygenic genetic effects, we first randomly selected 5,000 SNPs, assigning their main effects based on a normal distribution. These effects were then scaled to explain 5%, 30%, and 50% of the phenotypic variance, which corresponds to lower, median, and SNP heritability observed across 32 physical traits. In addition, we randomly selected 500 SNPs from odd-numbered chromosomes to simulate polygenic GxE effects, which allows us to use the even-numbered chromosomes to evaluate type I error of different methods. For each of the 500 selected SNPs, we determined the number of active environmental factors that it interacts with based on weighted sampling. Specifically, among 40 environmental factors, factors 1–10 were each assigned a weight of 10, while factors 11–40 were each assigned a weight of 1. Using these weights, we first sampled the number of active environmental factors for each SNP. Then, we selected the corresponding number of environmental factors without replacement. We simulated their GxE effect sizes from a standard normal distribution. Under the alternative settings, in addition to 500 SNPs with polygenetic GxE effects, we randomly selected one SNP and simulated GxE effects for power evaluation. We set the number of active environmental factors this SNP interacts with to be either 1, 2, 5, 10, 20, 30 or 40. We simulated its corresponding GxE effect sizes from a standard normal distribution and scaled them to account for either 0.1%, 0.12%, 0.14%, 0.16%, 0.18%, 0.2%, 0.22%, or 0.25% of phenotypic variance. We further scaled the polygenic GxE effects so that the total GxE effects, which included both the polygenic GxE effects and the GxE effect from the interacting SNP used for power evaluation, accounted for 5%, 15%, or 25% of the phenotypic variance. These values represent lower, median, and higher GxE heritability, as observed in the analysis of 32 physical traits. Finally, we simulated residual errors from a standard normal distribution and scaled them to account for the remaining proportion of phenotypic variance. We summed the GxE effect from the interacting SNP, the marginal effects of 40 environments, polygenic genotype effects, polygenic GxE effects, and residual errors to obtain individuals’ phenotypes.

In the simulations, we first created a baseline simulation scenario, where we set the number of active environments to be 30, the GxE effect of the SNP used for power evaluation to be 0.16% of the phenotypic variance, total SNP main effects to explain 30% of the phenotypic variance, and the total GxE effects to explain 15% of the phenotypic variance. We then modified one simulation parameter at a time on top of the baseline scenario to create 17 additional scenarios: six additional choices of the number of active environmental factors + seven additional choices of GxE effects + two additional choices of total SNP main effects + two additional choices of total GxE effects. Besides these scenarios, we created another six simulation scenarios on top of the baseline setting where we masked either 1, 2, 5, 10, 20, or 30 active environmental factors in the analysis, to evaluate the robustness of different methods when the environmental factors are not included in the analysis. For each simulation scenario, we performed 100 replicates. In the simulations, we evaluated type I error control using QQ plots and genomic inflation factor at different quantiles (0.5, 0.05, 0.01, 1e-3, and 1e-4) based on *p*-values from the even-numbered chromosomes across replicates. We assessed power by calculating the proportion of SNPs with true GxE effects that were successfully detected across replicates at a 1% family-wise error rate, which is achieved through Bonferroni correction adjusting for the number of SNPs. We also compared the power of fastGxE and fastGWA-GE in identifying the specific environmental factors driving the detected GxE associations. The power of fastGxE was assessed using a PIP threshold of > 0.5, while the power of fastGWA-GE was evaluated using a threshold of 0.05 divided by the total number of tests, calculated as the number of detected GxE associations multiplied by the number of environmental factors.

## Figures and Tables

**Table 1.** Summary of the detected GxE genomic loci for 32 physical traits.

Genomic locus	Gene	Trait	Lead SNP	*p*_main_	*p*_GxE_	Environment	Evidence
1:219449254-219515292	*LYPLAL1-AS1*	WHR adjusted for BMI	rs6674544	1.02E-45	3.81E-10	Sleeplessness; Processed meat; Alcohol	New
2:164645339-164752884	*COBLL1*	Waist/Hip Ratio	rs13389219	1.54E-29	1.73E-12	Using computer; Watching TV	New
		WHR adjusted for BMI	rs13389219	3.18E-48	2.83E-15	Using computer; Processed meat	New
3:41708768-42023513	*ULK4*	Pulse pressure	rs7647561	1.59E-44	3.19E-10	Age; Cheese	New
6:127118752-127208635	*RSPO3*	Waist/Hip Ratio	rs72961013	5.1E-108	3.80E-10	Smoking; Processed meat; Bread; Alcohol	New
		WHR adjusted for BMI	rs577721086	4.39E-188	3.73E-11	Bread	New
7:45921046-45970501	*IGFBP3*	Pulse pressure	rs11977526	1.51 E-38	1.02E-09	Age	New
7:121270692-121393067	*WNT16*	Heel bone mineral density	rs2908007	0	7.49E-15	Age	Known^13^
8:123581944-123596374	*FBXO32*	Pulse pressure	rs34557926	0.194711	7.19E-10	Age	New
15:78427159-78643826	*CHRNA5-A3-B4*	FEV1/FVC	rs8042849	0.0175437	3.98E-11	Watching TV; Smoking; Water	Known^13,18^
16:53763996-53811575	*FTO*	BMI	rs12149574	2.33E-143	8.38E-11	Stair climbing; Alcohol	Known^14,18^
		Weight	rs55872725	1.45E-139	1.62E-12	Age; Stair climbing; Alcohol	New
		Arm fat percentage	rs8063057	7.39E-112	4.58E-10	Age; Stair climbing; Alcohol	New
		Arm fat-free mass	rs56094641	4.5E-90	5.26E-13	Age; Stair climbing; Alcohol	New
		Arm predicted mass	rs56094641	7.89E-91	4.03E-13	Age; Stair climbing; Alcohol	New
		Basal metabolic rate	rs55872725	2.97E-110	5.08E-13	Age; Stair climbing; Alcohol	New
		Body fat percentage	rs12149574	8.46E-87	2.32E-11	Age; Stair climbing; Alcohol	New
		Hip circumference	rs62033400	1.17E-98	2.56E-10	Stair climbing; Alcohol	New
		Leg fat mass	rs12149574	6.15E-104	5.46E-10	Age; Alcohol	New
		Leg fat percentage	rs12149574	1.15E-81	6.28E-12	Age; Alcohol	New
		Leg fat-free mass	rs55872725	3.86E-123	2.43E-13	Stair climbing; Alcohol	New
		Leg predicted mass	rs55872725	1.28E-123	2.78E-13	Stair climbing; Alcohol	New
		Trunk fat-free mass	rs55872725	6.54E-73	5.95E-11	Age	New
		Trunk predicted mass	rs55872725	2.54E-72	9.99E-11	Age	New
		Waist circumference	rs55872725	2.4E-125	1.50E-13	Age; Stair climbing; Added salt; Alcohol	Known^18^
		Whole body fat mass	rs12149574	8.73E-103	1.01E-09	Age; Stair climbing; Alcohol	New
		Whole body fat-free mass	rs55872725	1.28E-99	1.49E-13	Alcohol	New
		Whole body water mass	rs55872725	3.34E-100	1.90E-13	Alcohol	New

The table lists summary information for each identified GxE genomic locus (rows), including genomic locus (chr: base pair position; 1^st^ column), the potential candidate GxE gene within the locus (2^nd^ column), associated trait (3^rd^ column), lead SNP (4^th^ column), *p*-value for SNP main effect (5^th^ column), *p*-value for GxE effect (6^th^ column), environmental factors contributing to the GxE signal (identified with posterior inclusion probability > 0.5; 7^th^ column), and evidence (New: potential novel discoveries; Known: previously identified loci; 8^th^ column).

**Table 2.** Summary of the detected GxE genomic loci for 67 blood biomarkers.

Genomic locus	Gene	Trait	Lead SNP	*p*_main_	*p*_GxE_	Environment	Evidence
1:21567383-21574964	*ALPL*	Alkaline phosphatase	rs3767145	1.18E-267	4.01E-10	Age	New
1:65427205-65749620	*LEPROT-LEPR*	C-reactive protein	rs1938498	0	1.18E-29	Age; Walking pace; Smoking; Processed meat; Bread	New
			rs55953331	0	7.38E-23	Phone use; Processed meat	New
			rs12409877	4.99E-208	2.48E-18	Smoking; Processed meat	New
1:109272258-109279544	*CELSR2*	LDL direct	rs7528419	0	6.02E-14	Age	Known^41^
		Apolipoprotein B	rs7528419	0	7.17E-14	Age; Alcohol	New
		Cholesterol	rs7528419	1.74E-235	2.17E-13	Age; Processed meat	Known^41^
1:159645742-159759257	*CRP*	C-reactive protein	rs3091244	0	4.87E-18	Age; Phone use; Hot drink; Bread	New	
			rs 11265263	0	1.39E-15	Age; Bread	New	
2:31387076-32813195	*SRD5A2*	Testosterone	rs138529890	4.5E-64	3.12E-13	Using computer; Processed meat; Bread	New	
2:210427857-211033748	*CPS1*	Glycine	rs1047891	0	0	Driving; Using computer; Getting up; Nap; Smoking; Processed meat; Bread; Alcohol; Friend visits	New
			rs34132242	0	6.36E-35	Driving; Smoking; Processed meat; Bread; Friend visits	New
			rs1990797	0	4.41E-31	Sleeplessness; Processed meat; Bread; Friend visits	New
			rs319737	0	3.38E-28	Driving; Using computer; Processed meat; Bread	New
			rs72941885	0	2.88E-25	Smoking; Processed meat; Bread	New
			rs111603337	0	2.25E-24	Driving; Processed meat; Bread	New
			rs4597454	0	6.53E-23	Driving; Processed meat; Bread; Alcohol	New
			rs34601425	0	3.38E-21	Nap; Processed meat; Bread	New
			rs12694211	0	5.47E-21	Driving; Nap; Processed meat; Bread	New
			rs79639185	0	3.31E-20	Processed meat; Bread; Alcohol	New
			rs62203749	6.49E-297	2.78E-16	Using computer; Processed meat; Bread	New
			rs 116277319	2.26E-198	6.12E-14	Driving; Processed meat	New
			rs13401425	0	7.68E-14	Driving; Processed meat; Bread; Friend visits	New
			rs62201973	1.58E-190	8.7E-13	Driving; Processed meat; Bread	New
			rs 114338044	2.82E-242	2.37E-11	Bread	New
2:233595452-233790330	*UGT1A1*	Direct bilirubin	rs887829	0	3.11E-56	Age; TDI; Watching TV; Walking pace; Sleeplessness; Smoking; Bread; Cereal; Water	New
		Total bilirubin	rs887829	0	2.55E-27	Age; Watching TV; Walking pace; Processed meat; Added salt; Bread; Tea; Water; Alcohol	New
3:56815721-56831748	*ARHGEF3*	Platelet distribution width	rs1354034	0.0886214	9.44E-16	Age; Watching TV; Water	Known^41^
4:9758866-10414736	*SLC2A9*	Urate	rs7696983	0	2.81E-59	Driving; Using computer; Nap; Sleeplessness; Processed meat; Raw vegetable; Bread; Friend visits	New
			rs10939663	0	8.7E-21	Driving; Processed meat; Bread; Friend visits	New
			rs4697948	2.79E-199	1.22E-19	Driving; Raw vegetable; Bread	New
			rs4461524	2.44E-270	4.96E-13	Driving; Processed meat; Bread; Friend visits	New
			rs6847871	0	1.36E-11	Driving; Processed meat; Friend visits	New
			rs1007872	2.22E-62	1.52E-11	Processed meat; Bread	New
6:25616225-26123274	*HFE*	Red blood cell distribution width	rs80215559	0	1.04E-09	Age; Alcohol	New
6:159844561-161022907	*LPA*	Lipoprotein A	rs6938647	0	2.52E-25	Age; Driving; Bread	New
			rs1510224	0	1.55E-15	Age; TDI; Driving	New
			rs3778217	4.16E-256	3.52E-14	Age; Dried fruit	New
			rs118039278	0	2.34E-12	Age	New
			rs113572208	0.680707	6.05E-11	Age; Using computer	New
9:133257521-133279427	*ABO*	Alkaline phosphatase	rs550057	0	1.89E-18	Watching TV; Walking pace; Alcohol	Known^33^
10:63063068-63640592	*JMJD1C*	Testosterone	rs9414801	1.18E-176	2.2E-30	Driving; Processed meat; Bread; Alcohol; Confide	New
10:112989975-113058995	*TCF7L2*	Glycated haemoglobin (HbA1c)	rs7903146	7.53E-139	1.06E-09	Age	Known^41^
11:14557830-14913140	*CYP2R1*	Vitamin D	rs116970203	0	1.67E-16	Age; TDI; Moderate PA; Computer games	New
			rs10766194	0	2.78E-16	Age; TDI; Moderate PA; Vigorous PA	New
11:116752497-116786845	*BUD13*	LDL direct	rs964184	1.17E-60	8.06E-13	Age; Processed meat	New
12:2799164-2868788	*FKBP4*	Testosterone	rs56196860	9.2E-131	7.91E-27	Driving; Processed meat; Bread	New
12:120946692-121047133	*HNF1A*	C-reactive protein	rs2244608	0	1.55E-15	Age	New
14:94371805-94378610	*SERPINA1*	Testosterone	rs28929474	2.32E-39	9.99E-16	Age; Using computer; Bread	New
16:20339137-20395874	*UMOD*	Creatinine	rs28640218	9.07E-112	8.33E-16	Age	New
		Cystatin C	rs13333226	3.58E-76	1.39E-15	Age	New
		Urea	rs9928757	1.44E-13	2.99E-10	Age	New
17:7406687-7748588	*NLGN2*	Testosterone	rs62059839	0	8.95E-66	Age; Vigorous PA; Driving; Nap; Sleeplessness; Processed meat; Bread; Alcohol; Friend visits	New
			rs9892862	5.22E-303	1.6E-49	Age; Vigorous PA; Driving; Using computer; Sleeplessness; Processed meat; Bread; Alcohol; Friend visits	New
			rs1042522	3.32E-203	1.41E-32	Getting up; Processed meat; Bread; Alcohol; Friend visits	New
			rs12944954	3.54E-152	4.13E-28	Age; Using computer; Processed meat; Bread	New
			rs3174744	3.84E-117	1.89E-19	Processed meat; Bread	New
			rs117387630	2.75E-44	9.84E-13	Nap; Bread	New
			rs113924794	5.83E-60	5.61E-12	Using computer; Walking pace; Processed meat; Bread	New
			rs55662831	5.91 E-75	9.84E-12	Driving; Processed meat	New
		SHBG	rs115126578	0	1E-14	Getting up; Processed meat; Raw vegetable	New
			rs858519	0	9.08E-10	Processed meat	New
19:11075243-11100236	*LDLR*	LDL direct	rs73015024	0	2.76E-14	Age; Smoking	Known^41^
		Cholesterol	rs73015024	0	1.25E-13	Age	Known^41^
		Apolipoprotein B	rs73015024	0	1.4E-13	Age; Walking pace	New
19:19215154-19682736	*NCAN*	Apolipoprotein B	rs8107974	9.88E-91	1.11E-20	Age; Sleeplessness; Processed meat; Raw vegetable	Known^33^
		Cholesterol	rs58542926	2.36E-145	2.94E-15	Sleeplessness; Oily fish; Processed meat	New
		LDL direct	rs58542926	4.52E-120	3.94E-15	Sleeplessness; Oily fish; Processed meat; Raw vegetable	New
		Aspartate aminotransferase	rs8107974	1.4E-34	4.93E-10	Vigorous PA	New
19:44728895-44923868	*APOE*	LDL direct	rs7412	0	2.74E-27	Age; Watching TV	Known^41^
			rs429358	0	5.71E-21	Age; Bread	
		Cholesterol	rs7412	0	1.53E-25	Age; Watching TV	Known^41^
			rs429358	0	1.67E-16	Age; Smoking; Bread	
		C-reactive protein	rs429358	0	8.33E-16	Age	New
		Apolipoprotein B	rs429358	0	1.55E-15	Age	New
			rs7412	0	7.49E-15	Age	New
		Triglycerides	rs769449	1.49E-56	2.54E-10	Age	Known^33^
22:24590177-24608641	*GGT1*	Gamma glutamyltransferase	rs2003757	0	4.76E-39	Age; Driving; Processed meat; Bread	New
22:43928847-43999571	*PNPLA3*	Aspartate aminotransferase	rs3747207	4.12E-220	2.01E-25	Watching TV; Walking pace; Alcohol	New
		Alanine aminotransferase	rs3747207	9.47E-199	2.25E-14	Age; Smoking	New

The table lists summary information for each identified GxE genomic locus (rows), including genomic locus (chr: base pair position; 1^st^ column), the potential candidate gene within the locus (2^nd^ column), associated trait (3^rd^ column), lead SNP (4^th^ column), *p*-value for SNP main effect (5^th^ column), GxE *p*-value (6^th^ column), environmental factors contributing to the GxE signal (identified with posterior inclusion probability > 0.5; 7^th^ column), and evidence (New: potential novel discoveries; Known: previously identified loci; 8^th^ column).
